# NATIONAL IMPLEMENTATION OF A VHA DIFFUSION OF EXCELLENCE GOLD STATUS PRACTICE

**DOI:** 10.1093/geroni/igz038.1772

**Published:** 2019-11-08

**Authors:** Kimberly K Garner, Jamie Jensen, Lisa Nabholz, Melissa Harding, Laura Taylor

**Affiliations:** 1 VISN 16/Little Rock Geriatric Research Education Clinical Center - Department of Veteran Affairs, Little Rock, Arkansas, United States; 2 National Social Work Program Office - Department of Veteran Affairs, Washington, D.C., United States

## Abstract

Advance care planning (ACP) is a health behavior that requires in-depth discussions support by trained professionals and motivational strategies to promote goal-setting and actions. Group visits in the healthcare setting can effectively increase an individual’s motivation and self-efficacy for ACP. The Department of Veterans Affairs (VA) has developed the Diffusion of Excellence Initiative to identify and spread innovative practices such as Advance Care Planning via Group Visits (ACP-GV), which uses an interactive group session to engage Veterans in thinking about and planning for their future medical decisions. In these sessions, social workers, nurses, psychologists, and chaplains, facilitate group discussions to increase the chance that a Veteran’s care preferences are known and reflect with their wishes. This also can relieve trusted others of having to make these tough decisions without much guidance. In addition, ACP-GV increases the effectiveness of advance care planning through allowing Veterans to discuss and process this complex topic with their peers. To date, 36 VA Medical Centers (VAMCs) are currently adopting, implementing or sustaining this practice and more than 15,250 Veterans have attended ACP-GV sessions. In addition, another 40 VAMCs are exploring participating this practice. Of those participants, approximately 18-20% develop a new advance directive and 86% set a smart goal to take steps toward advance care planning. Continued dissemination and implementation of this innovative practice is ongoing. After the session, attendees will have practical guidance for implementation of ACP-GV discussions in integrated (VA) or fee-for-service (Medicare) settings.

